# Application of Alkali Lignin and Spruce Sawdust for the Effective Removal of Reactive Dyes from Model Wastewater

**DOI:** 10.3390/molecules28104114

**Published:** 2023-05-16

**Authors:** Kateřina Hájková, Michaela Filipi, Roman Fojtík, Ali Dorieh

**Affiliations:** 1 Department of Wood Processing and Biomaterials, Faculty of Forestry and Wood Sciences, Czech University of Life Science Prague, Kamýcká 129, 165 00 Prague, Czech Republic; fojtikr@fld.czu.cz (R.F.); dorieh@fld.czu.cz (A.D.); 2 Institute of Chemistry and Technology of Macromolecular Materials, Faculty of Chemical Technology, University of Pardubice, Studentská 572, 532 10 Pardubice, Czech Republic; michaela.filipi@upce.cz

**Keywords:** alkali lignin, spruce sawdust, reactive dyes, decolorization

## Abstract

Today, the emphasis is on environmentally friendly materials. Alkali lignin and spruce sawdust are suitable natural alternatives for removing dyes from wastewater. The main reason for using alkaline lignin as a sorbent is the recovery of waste black liquor from the paper industry. This work deals with removing dyes from wastewater using spruce sawdust and lignin at two different temperatures. The decolorization yields were calculated as the final values. Increasing the temperature during adsorption leads to higher decolorization yields, which may be due to the fact that some substances react only at elevated temperatures. The results of this research are useful for the treatment of industrial wastewater in paper mills, and the waste black liquor (alkaline lignin) can be used as a biosorbent.

## 1. Introduction

There has been a growing worldwide interest in environmental issues related to emissions in recent years. This is because pollutants that affect water, soil, and air are constantly released into the environment, producing many residues. One of the most critical industries that is essentially considered a source of water pollution is the dye industry, whose products are used in many applications such as textiles, paper and pulp, wood composites, cosmetics, soap, colored glass, paints, ceramics, polymers, adhesives, art supplies, beverages, wax, and biomedicines [1,2,3].

The world’s yearly production of dyes is estimated to be about 7 × 10^5^ tons, and approximately 15% of this is released into the environment as liquid waste [4]. The producers and consumers of dyes are interested in the dye’s stability and permanence and produce shades that do not easily disintegrate after use [5]. Due to the volume and complexity of its wastewater, the textile industry is among the most polluting treatments [6]. From the total worldwide consumption of dyes in the textile industry, it is approximated that 90% ends up in the fabrics [7]. Inefficiency in the dyeing of textiles leads to enormous amounts of dye that off-track directly to wastewater and harm flora and fauna [8,9,10,11]. Acid dyes are organic sulfonic acids; the commercially available forms are usually sodium salts, showing good water solubility [10]. Acid dyes are primarily effective on certain fiber types, such as wool, silk, and polypropylene fibers, and blends, such as cotton.

Reactive acid dyes, which contain a reactive functioning group such as chlorotriazine, are azo dyes or azo compounds associated with a metal complex and interact with cotton and wool to form a covalent bond. Reactive dyes hydrolyze easily, which presents another application problem. The hydrolyzed dye does not react with the substrate to form a covalent bond but stays in the dye bath, polluting the wastewater. Due to the high aromatic content of dye molecules and the stability of modern dyes, biological treatment is inefficient for their degradation. Many processes are available for the disposal of dyes using conventional treatment technologies, including biological and chemical oxidation, chemical coagulation, foam flotation, electrolysis, biodegradation, advanced oxidation, photocatalysis, and membrane adsorption processes [10,12,13,14].

Various adsorbents have been used to remove dyes from aqueous solutions [15,16,17,18]. The application of Aqai stalks (*Euterpe oleracea*) to remove textile dye from aqueous solutions was studied regarding the effect of pH, biosorbent doses and shaking time, and nitrogen adsorption–desorption curves [15]. The removal of reactive dye from wastewater using Brazilian pine (*Araucaria angustifolia*) shell was favorable at pH values around 2–7 [16]. Cupuassu (*Theobroma grandiflorum*) shell as a sorbent in the acidic pH region showed favorable biosorption of dyes [17]. Spirulina platensis microalgae were tested as a sorbent in industrial wastewater and were able to remove 94.4–99.0% of the dye from mixtures.

Zaidi et al. [19] investigated the use of *Artocarpus odoratissimus* leaves to remove malachite green dye from simulated wastewater. They demonstrated high adsorption efficiency with a maximum adsorption capacity of 254.93 mg g^−1^. Compared to other adsorbents, the leaves of *Artocarpus odoratissimus* showed little effect on pH changes, supporting its potential for use in wastewater treatment.

The abundantly available *kangkong* root can be used to remove dyes; Lu et al. [20] used it to remove methyl violet. They were primarily concerned with the recovery and reuse of the dye and concluded that sodium-hydroxide-modified *kangkong* root could sustain dye removal after five consecutive cycles.

**Figure 1:**
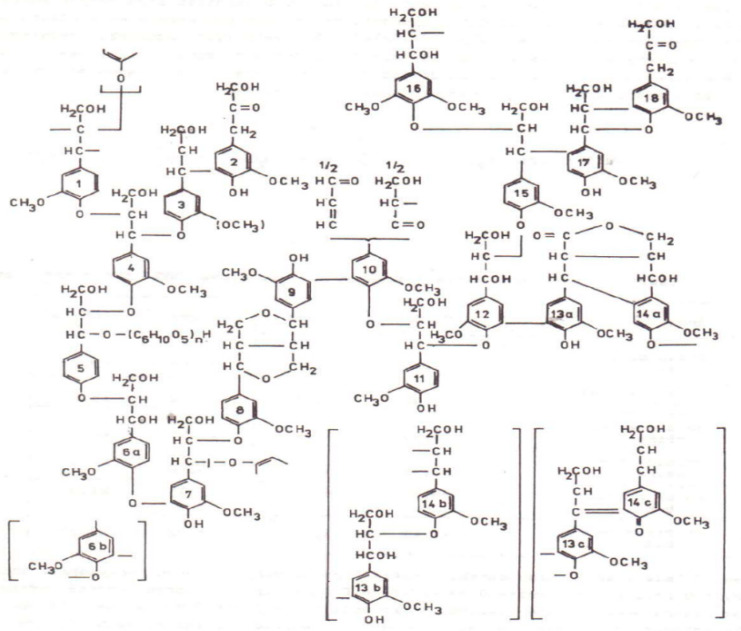
Scheme of softwood lignin [23].

Lignin is a broad term for a large group of aromatic polymers formed by the combinatorial oxidative coupling of 4-hydroxy phenylpropanoids (Figure 1). The elemental composition and methoxyl content of lignin in spruce wood is 63.84% carbon, 6.04% hydrogen, 29.68% oxygen, and 15.75% methoxyl [19,20].

These polymers are mainly deposited in the walls of secondarily thickened cells, making them rigid and impermeable [21]. Lignin is one of the three main components of lignocellulosic biomass and is a renewable raw material with great potential [22]. Lignin is contained in the cell walls of almost all terrestrial plants. Lignin is the second most plentiful component in vegetation, surpassed only by cellulose. It is a polymer that does not consist of carbohydrate monomers but contains many aromatic compounds. Depending on the species, it contains up to three different phenylpropane monomers in different proportions. Coniferyl alcohol occurs in all species and is the dominant monomer in conifers.

Currently, the global production of technical lignin is approximately 100 million tons/year [24,25], with kraft-processed softwood lignin being the most produced technical lignin after lignosulfonate [26,27,28].

Materials such as activated carbon, rice husk, cotton seed shell, and oak wood waste are commonly used for dye removal. Activated carbon is a commercial adsorbent for eliminating pollution from wastewater. However, activated carbon’s higher production cost and regeneration difficulty limit its widespread use [29]. Thus, developing cheaper, eco-friendly, and more efficient adsorbents is a subject of intensive research.

Sawdust is widely available in the timber and forest industries and has recently been studied as an adsorbent. Sawdust materials are biodegradable and have an affinity for water. The wood powder does not noticeably or appreciably decompose on prolonged contact with water [30]. Due to its lignocellulosic composition, sawdust can be a cheap adsorbent [31,32]. As most spruce monoculture is planted in Central Europe and is subsequently used for paper production, spruce sawdust supplied directly from the paper mill was used for the analysis.

This study was carried out to remove reactive dyes from model effluents. The research focused on two areas. The first was the sorption capacity of dye concentration at 25 and 90 °C. The second was the sorbent dosage at 25 and 90 °C. Since dye removal is essential for paper mills, material commonly found in paper mills, or in the case of black liquor (alkaline lignin), even waste material, was used.

## 2. Results and Discussion

**Figure 2:**
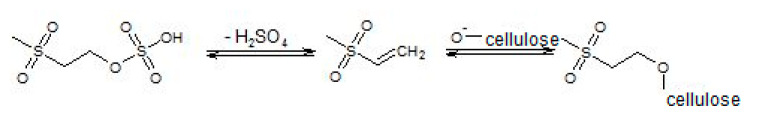
Elimination mechanism dyes [34].

This work focused on eliminating reactive dyes from model wastewater through chemisorption (Figure 2). Cheap lignocellulosic waste is a simple source of OH nucleophilic groups [33] that can be used for the chemical extraction of reactive dyes.

**Figure 3:**
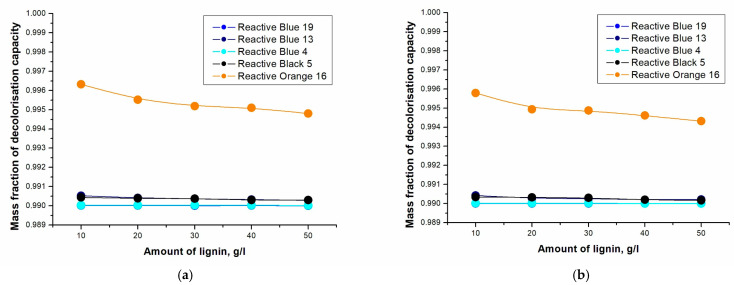
Dependence of the decolorization capacity on the amount of alkaline lignin: (**a**) unheated sample (25 °C); (**b**) heated sample (90 °C).

**Figure 4:**
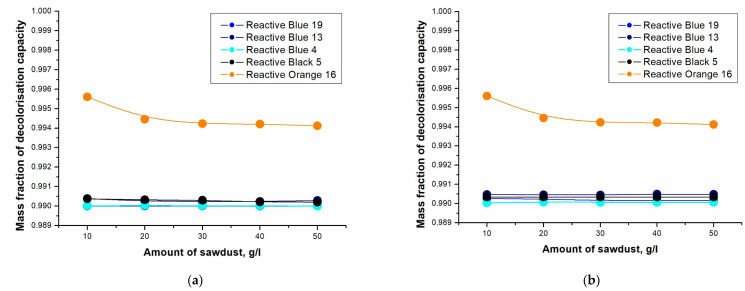
Dependence of the decolorization capacity on the amount of spruce sawdust: (**a**) unheated sample (25 °C); (**b**) heated sample (90 °C).

The effect of different sorbents on dye chemisorption was investigated. The results for each sorbent are shown in Figure 3 and Figure 4. Table 1 shows the decolorization equations for the individual dyes using spruce sawdust and alkaline lignin as sorbents.

Figure 3 and Figure 4 show that the highest removal of dye was for the reactive orange 16 dye. To remove this dye, alkaline lignin was more effective, but the result of spruce sawdust as a sorbent was also very high. The sorbent concentration for alkaline lignin was 10 g∙L^−1^, and in the case of spruce sawdust, only 0.5 g∙L^−1^ was sufficient. The values for the other dyes were very similar; see Table 1.

In Table 1, it can be seen from the decolorization equations that the dye reactive orange 16 was indeed the most captured. For reactive blue 13, higher decolorization occurred with spruce sawdust, similar to reactive blue 4 and reactive black 5. Reactive blue 19 had the same decolorization capacity when using alkaline lignin and spruce sawdust as sorbents. The decolorization results were very high for both sorbents used, so it is not possible to conclude whether it is preferable to use spruce sawdust or alkaline lignin.

**Figure 5:**
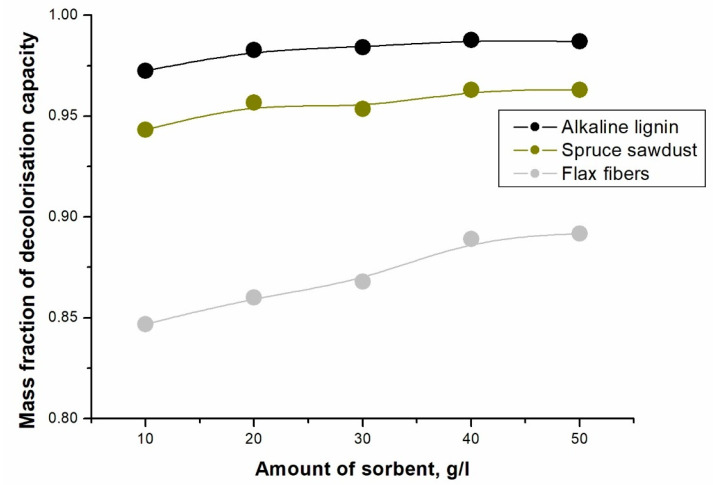
Dependence of decolorization capacity on sorbent loading at 70 °C.

The increasing chemisorption yield with biomaterial dose can be attributed to the increased surface area and the availability of more binding sites of the biosorbent. Studying the temperature dependence of the adsorption reactions provides insight into the changes during adsorption [35,36]. For this reason, the adsorption was higher for the heated sorbent. Adsorption is endothermic, with adsorption capacity increasing with increasing temperature. An increase in temperature can cause an increase in the endothermic adsorption, the mobility of dye molecules, and the number of active sites for adsorption [37]. For dyes and sorbents, specific structural changes may occur at elevated temperatures [38]. Water molecules displaced by the adsorbate gain more translational entropy than the adsorbate molecules lose, allowing randomness to dominate the system [39]. However, when attractive hydration forces prevail, an exothermic process associated with a decrease in entropy occurs. The increase in adsorption suggests that adsorption was already rapid at low adsorbent doses [40]. The reactive orange 16 dye was tested for comparison and achieved the best decolorization—Figure 5. Spruce sawdust and alkaline lignin were supplemented with another biomass-based material, namely flax, as adsorbents.

Figure 5 shows that lignin was the most reactive sorbent for the most reactive dye, reactive orange 16, at a suspension temperature of about 70 °C. However, a very high decolorization result was also achieved for spruce sawdust. For comparison, flax fiber with the same weight as alkaline lignin and spruce sawdust was used, but the flax fiber did not show the same discoloration as spruce sawdust and alkaline lignin. This may be due to the chemical composition of the flax. Since spruce sawdust contains significantly more lignin than flax, this chemical component may be the appropriate sorbent.

## 3. Materials and Method

### 3.1. Materials

#### 3.1.1. Spruce Sawdust

The spruce sawdust was milled using a pulp milling process from Mondi Štětí (Štětí, Czech Republic), a paper company that deals with kraft pulp cooked mainly from spruce monoculture. The dry matter content of the spruce sawdust was 95%. For the analysis, spruce sawdust samples were ground using a knife mill for about 20 s with an IKA MF 10 BASIC (Staufen, Germany) and then dried to a constant weight at 105 °C according to the Tappi T210 cm-03 standard [41].

#### 3.1.2. Black Liquor

Black liquor from the flax biorefining process was obtained from Delfort Olšany, Czech Republic (dry matter content 42 wt.%). This paper production is based on the soda process, so the black liquor contains sodium hydroxide.

The black liquor’s properties were analyzed and are shown in Table 2 to compare the variables that enter and exit the production. Therefore, physical quantities such as pH, density, viscosity, interfacial tension, and alkaline lignin concentration were analyzed.

Alkaline lignin from black liquor was obtained by precipitation with sulfuric acid. Specifically, sulfuric acid was added to the black liquor in a slight excess, causing an exothermic reaction. Subsequently, the mixture was allowed to react, and after precipitation, the already solid alkali lignin was processed using a friction pan to powder material.

**Figure 6:**
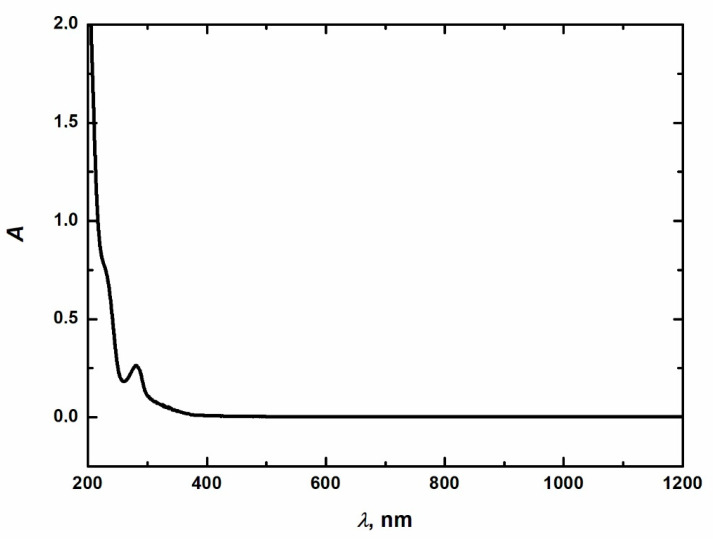
Absorption spectrum of alkaline lignin.

The alkaline lignin and sodium concentrations in black liquor were 45 g·dm^−3^ and 39 g·dm^−3^, respectively. The other properties of the black liquor were as follows: dry matter content 17.1%, of which ash constituted 65% and organic substances were 35%, and chemical oxygen consumption ρ(O_2_) = 170 g·dm^−3^. The absorption spectrum of alkaline lignin was measured on a GBC Cintra 10e spectrophotometer (GBC, Braeside, Australia). Based on the measurement of the absorption spectrum of alkali lignin, a wavelength of 280 nm was chosen—Figure 6.

**Figure 7:**
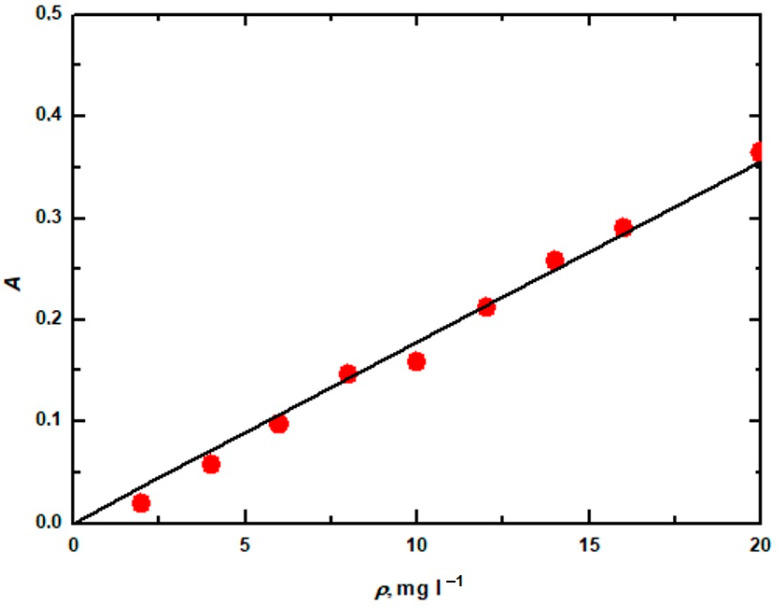
Spectrophotometer calibration dependence for alkali lignin at a wavelength of 280 nm.

The immediate solution of alkaline lignin was prepared by dissolving 100 mg of alkaline lignin in 1 L of aqueous NaOH solution with a concentration of 1 mol∙L^−1^. The other solutions were prepared by dilution. They were found to absorb at a wavelength of 280 nm. These absorbances are shown in the graph in Figure 7. This dependence determined the concentration of alkali lignin in the sodium hydroxide solution. The reliance was derived based on calibrating the measured values with a spectrophotometer. The dependence of the absorbance *A* on the concentration of alkali lignin ρ (g∙L^−1^) is shown by the equation:

*A* = 16.923*ρ*. (1)

#### 3.1.3. Dyes

Merck reactive dyes were used for the experiments. Their specifications are listed in Table 3.

### 3.2. Experimental Methods

Aqueous solutions of the dyes were prepared by dissolving the respective dye in distilled water [42]. Spruce sawdust and alkaline lignin, precipitated from black liquor as already mentioned, were used as sorption materials. The dyes were mixed at different concentrations, and their absorbance was measured. The concentration–absorbance relationship of each dye was plotted on graphs and interspersed with calibration curves from which the ideal concentration for the experiment was evaluated, Table 4.

The dyes, at the concentrations shown in Table 4, were dissolved in a volume ratio of 1:24 in distilled water. The solutions thus prepared were mixed with alkaline lignin made from black liquor and then dissolved; the alkaline lignin was dosed in weights of 5, 10, 15, 20, and 25 g. Similar to the alkaline lignin, another batch of the solutions was mixed with 5, 10, 15, 20 and 25 g of spruce sawdust. The pH value was then adjusted to pH = 12 using sodium carbonate. The carbonate mixture was stirred vigorously for two hours at a laboratory temperature of 25 °C (unheated samples) and 90 °C (heated samples). After two hours of stirring, the pH of the suspension was acidified with 16% sulfuric acid.

The suspension volume was made up to 500 mL using distilled water, thus obtaining a sample for filtration. The filtered samples were analyzed using a spectrophotometer, and the dye concentration was calculated from the absorbance of the suspension [43,44,45]. Absorbance measurements were always carried out against a reference sample.

## 4. Conclusions

Wastewater treatment, especially in the paper industry’s deinking area, is a topical issue; dyes must be removed, and biobased material should be used as the sorbent. Both alkaline lignin and spruce sawdust are materials that belong to the biomaterial category and have the advantage of being used in other operations in paper mills, so they are materials commonly used in paper mills.

The decolorization equation states that the two sorbents provide almost comparable decolorization even at a small sorbent dose in the effluent. Better values were obtained at elevated temperatures, but the results were favorable even at room temperatures.

The sorbents we tested were compared with flax fiber, which did not show the same decolorization capacity as alkaline lignin and spruce sawdust. This is probably due to the lower lignin content in annual plants.

Of the sorbents used, the most effective was alkaline lignin, obtained from black liquor, a waste from pulp cooking.

Therefore, alkaline lignin extracted from papermaking waste is a suitable alternative sorbent for industrial wastewater treatment.

## Figures and Tables

**Table 1:** Decolorization equation.

Sorbent	Dyes	Decolorization Equation
Alkaline lignin unheated sample	Reactive blue 13	X = 0.9803ρ + 0.0036
	Reactive blue 19	X = 0.9900ρ + 0.0002
	Reactive blue 4	X = 0.9801ρ + 0.0004
	Reactive black 5	X = 0.9804ρ + 0.0026
	Reactive orange 16	X = 0.9945ρ + 0.0203
Alkaline lignin heated sample	Reactive blue 13	X = 0.9902ρ + 0.0025
	Reactive blue 19	X = 0.9900ρ + 0.0001
	Reactive blue 4	X = 0.9900ρ + 0.0001
	Reactive black 5	X = 0.9901ρ + 0.0036
	Reactive orange 16	X = 0.9940ρ + 0.0202
Spruce sawdust unheated sample	Reactive blue 13	X = 0.9903ρ + 0.0000
	Reactive blue 19	X = 0.9900ρ + 0.0000
	Reactive blue 4	X = 0.9900ρ + 0.0000
	Reactive black 5	X = 0.9902ρ + 0.0001
	Reactive orange 16	X = 0.9938ρ + 0.0008
Spruce sawdust heated sample	Reactive blue 13	X = 0.9905ρ + 0.0000
	Reactive blue 19	X = 0.9901ρ + 0.0000
	Reactive blue 4	X = 0.9901ρ + 0.0000
	Reactive black 5	X = 0.9903ρ + 0.0000
	Reactive orange 16	X = 0.9938ρ + 0.0008

**Table 2:** The properties of black liquor.

Properties of Black Liquor	Amount in Black Liquor
Total dry matter content, %	42.0
Ash content of total dry matter, %	27.3
Content of organic substances from total dry matter, %	14.7
Concentration of alkali lignin, g∙dm^−3^	45.0
Sodium concentration, g∙dm^−3^	39.0
Chemical oxygen consumption ρ(O_2_), g∙dm^−3^	170.0
Density, kg∙m^−3^	1062.0
Viscosity, mPa∙s	1.4
Surface tension, mN∙m^−1^	55.2
pH value	12.1

**Table 3:** Characterization of reactive dyes.

Reactive blue 13	Dye content 100 wt.%, A_max_ = 682 nm	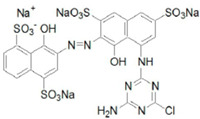
Reactive blue 19	Dye content 50 wt.%, A_max_ = 684 nm	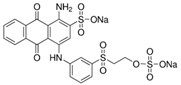
Reactive blue 4	Dye content 35 wt.%, A_max_ = 595 nm	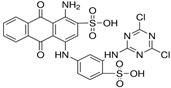
Reactive black 5	Dye content 50 wt.%, A_max_ = 597 nm	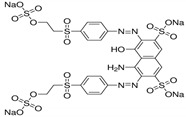
Reactive orange 16	Dye content 70 wt.%, A_max_ = 494 nm	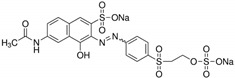

**Table 4:** Concentration of dyes.

Dyes	Concentration, mol∙L^−1^	Calibration Curve Equation
Reactive blue 13	0.01	*ρ =* 8317.7*A* + 0.184
Reactive blue 19	0.01	*ρ =* 11,686*A* + 0.498
Reactive blue 4	0.01	*ρ =* 20,886*A* + 0.511
Reactive black 5	0.01	*ρ =* 2712*A* + 0.019
Reactive orange 16	0.007	*ρ =* 2086.6*A* + 0.435

## Data Availability

Data are available on request due to ethical restrictions. The data presented in this study are available on request from the corresponding author. The data are not publicly available due to (unfinished research).
